# Hepatitis A: Epidemiology, High-Risk Groups, Prevention and Research on Antiviral Treatment

**DOI:** 10.3390/v13101900

**Published:** 2021-09-22

**Authors:** Marion Migueres, Sébastien Lhomme, Jacques Izopet

**Affiliations:** 1 Virology Laboratory, Hôpital Purpan, CHU Toulouse, 31300 Toulouse, France; lhomme.s@chu-toulouse.fr (S.L.); izopet.j@chu-toulouse.fr (J.I.); 2 Institut Toulousain des Maladies Infectieuses et Inflammatoires (Infinity), INSERM UMR1291-CNRS UMR5051, 31300 Toulouse, France; 3 Université Toulouse III Paul-Sabatier, 31062 Toulouse, France

**Keywords:** hepatitis A, high-risk groups, men who have sex with men, people who use substances, homelessness, vaccination, epidemiology

## Abstract

The hepatitis A virus (HAV) is a leading cause of acute viral hepatitis worldwide. It is transmitted mainly by direct contact with patients who have been infected or by ingesting contaminated water or food. The virus is endemic in low-income countries where sanitary and sociodemographic conditions are poor. Paradoxically, improving sanitary conditions in these countries, which reduces the incidence of HAV infections, can lead to more severe disease in susceptible adults. The populations of developed countries are highly susceptible to HAV, and large outbreaks can occur when the virus is spread by globalization and by increased travel and movement of foodstuffs. Most of these outbreaks occur among high-risk groups: travellers, men who have sex with men, people who use substances, and people facing homelessness. Hepatitis A infections can be prevented by vaccination; safe and effective vaccines have been available for decades. Several countries have successfully introduced universal mass vaccination for children, but high-risk groups in high-income countries remain insufficiently protected. The development of HAV antivirals may be important to control HAV outbreaks in developed countries where a universal vaccination programme is not recommended.

## 1. Background and Virology

The hepatitis A virus (HAV) belongs to the *Hepatovirus* genus within the *Picornaviridae* family [1]. There are two types of infectious HAV particles: naked and quasi-enveloped virions. Quasi-enveloped virions have a lipid membrane and are found in the blood and culture supernatants [2]. Naked virions are quasi-enveloped virions in which the membrane has been removed by the detergent action of bile acids within the biliary canaliculus before they are excreted in the faeces [3]. HAV has a single positive-strand 7.5 kb RNA genome with a single open reading frame (ORF) encoding one large polyprotein [4]. This polyprotein is processed by viral (protease 3C) and host cell proteases to give the structural (VP4, VP2, VP3, and VP1) and the non-structural mature proteins (2B, 2C, 3A, 3B, 3C (protease), and 3D (RNA-dependent RNA polymerase)) [5,6,7]. Based on the latest International Committee on Taxonomy of Viruses (ICTV) report, HAV is now classified into five genotypes [8]. Among them, only genotypes I, II, and III, further divided into subtypes A and B, infect humans. A third subgenotype, named IC, has been proposed for genotype I but is not yet recognized by the ICTV [9]. There is only one serotype despite the existence of several genotypes.

## 2. Clinical Outcomes

The 4-week initial incubation period is often followed by a nonspecific prodromal phase during which a person suffering from infection can experience flu-like syndrome and intestinal disorders for a few days. The next, icteric, phase is defined by jaundice and hepatic cytolysis with elevated serum aminotransferase activities [10,11]. While infection is largely asymptomatic in children (>90% of children less than 6 years old), symptoms are much more common (>70%) in adults [12,13]. Older patients are at increased risk of severe outcomes, hospitalization, and death [14,15]. Fulminant hepatitis is rare, occurring in less than 1% of cases, but cholestatic forms and relapsing hepatitis have also been described [16]. Relapsing hepatitis occurs in about 3–20% of patients, usually 3 to 12 weeks after the initial episode, but the symptoms are less severe than the initial ones [17]. HAV does not cause chronic infections, unlike other hepatitis viruses. Extra-hepatic manifestations of acute hepatitis A are rare but can include neurological symptoms such as Guillain–Barre syndrome, rash, pancreatitis, arthritis, myocarditis, acute kidney injury, and haematological disorders such as haemolysis and cryoglobulinemia [18,19,20,21].

## 3. Hepatitis A Diagnosis

Biological diagnosis is required because hepatitis A is clinically indistinguishable from other viral forms of hepatitis. Acute hepatitis A is mainly diagnosed by demonstrating anti-HAV IgM. Anti-HAV IgM antibodies appear a few days before or concurrently with the onset of clinical symptoms. Their titre remains high for about 1 month and then gradually decreases to zero over about 6 months in most patients [6,22]. False-positive results can occur due to specificity problems, and anti-HAV IgM can also be detected following vaccination. Therefore, this analysis should only be conducted when it is clinically suspected [23]. Anti-HAV IgG antibodies appear soon after the IgM antibodies and persist for many years, conferring lifelong immunity. Their presence indicates past, resolved infections [13,24].

Nucleic acid amplification tests are rarely used for diagnosis, but they can detect HAV RNA in the faeces and plasma of patients who have been infected [25,26,27] and in contaminated water and food [28]. Sequencing and phylogenetic analysis are mainly used to track outbreaks, and these techniques are particularly useful for identifying transmission routes [6].

## 4. Transmission Routes of Hepatitis A Virus and Epidemiology

HAV is highly resistant to harsh physical conditions such as high ambient temperatures, acidity, and freezing for several hours or even several months [29,30,31]. Its high resistance is due to its folding-dependent, highly cohesive capsid [32]. This physical resistance plus the shedding of high titres of the virus in the faeces of individuals who have been infected explains why transmission is linked to poor hygiene and contamination of waste water when sanitation is suboptimal [33]. This mainly faecal–oral pattern of transmission can be direct through contact with an individual who has been infected or indirect by ingestion of contaminated water or food. Person-to-person infections are responsible for the majority of outbreaks in developed countries, whereas foodborne infections generally lead to sporadic cases. However, several fairly recent outbreaks in developed countries have been linked to contaminated food [34,35]. Seafood represents one of the main source of infection [36,37], but more and more outbreaks now involve imported frozen food, such as frozen berries and vegetables, and ready-to-eat foods [38]. Due to the viraemic phase in patients who have been infected, bloodborne infections can also occur in blood transfusion recipients, although such cases are rare. Frequent recipients of blood products, such as patients with haemophilia, were long considered to be at increased risk of infection and were recommended for vaccination. However, improvements in virus inactivation, the use of sterilized recombinant clotting factors, and the screening of plasma for HAV in the US have ensured that these patients are now no more at risk than the general population [24]. The virus can also be transmitted during organ transplantation [39].

There are estimated to be 100 million HAV infections and 1.5 million symptomatic cases annually worldwide, and these are responsible for 15,000 to 30,000 deaths per year [22]. A recent publication based on data taken from the Global Burden of Disease (GBD) 2019 database found that hepatitis A ranked first in terms of incidence rate among the four major acute forms of viral hepatitis (A, B, C, and E). The worldwide age-standardized incidence rate remained stable between 1990 and 2019, while there was a significant decrease in the age-standardized disability-adjusted life years (DALYs) [40,41]. The incidence of hepatitis A varies considerably between countries depending on the socio-demographic index [41]. Low- and middle-income countries, mainly in Africa and South Asia, have the greatest hepatitis A burdens [13,42]. The HAV infection burden is classically determined by surveys to detect anti-HAV immunoglobulin G, a marker of past infection [43]. The endemic nature of HAV is classified according to age-specific prevalence into high (≥90% by age 10 years), intermediate (≥50% by age 15 years with <90% by age 10), low (≥50% by age 30 with <50% by age 15), and very low (≤50% by age 30) [22]. The frequency of HAV infections in countries with low hygiene standards and poor socioeconomic conditions means that people are exposed to the virus early in life, resulting in frequent asymptomatic infections and a high proportion of immune adults. In contrast, the lower exposure to the virus in countries with higher sanitary and socioeconomic conditions leads to a greater proportion of susceptible individuals and adults who are at increased risk of symptomatic, severe disease. This “epidemiologic paradox” is particularly concerning for countries that are developing their water and sanitation systems and expanding their economy. Infection rates remain low in areas where HAV is rare due to poor HAV circulation despite low immunity rates as long as the disease is not introduced from an external source [22]. Some outbreaks have occurred, mainly in individuals at specific risk. Globalization, with increased travel and food shipments, could lead to changes in the epidemiology of hepatitis A [42].

## 5. Specific Risk Groups in Developed Countries

**Figure 1:**
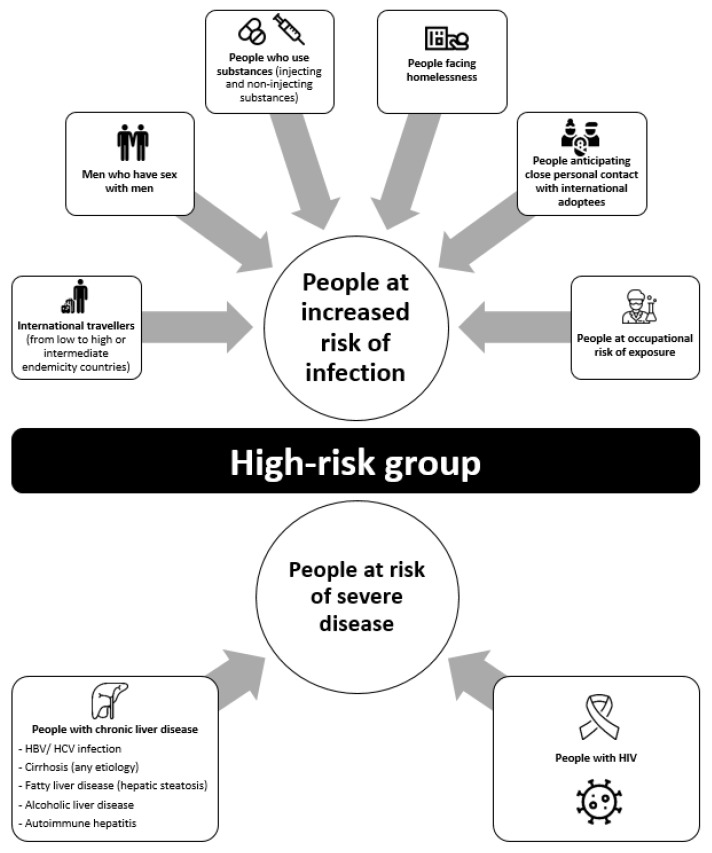
High-risk group vaccination recommendations [24].

Most clusters of HAV infections in developed countries, where most adults are susceptible, are due to person-to-person transmission among high-risk groups. Surveillance networks have been used to identify specific populations at increased risk of HAV infections or those at increased risk of fulminant hepatitis. Current guidelines recommend HAV vaccination for these specific groups [24] (Figure 1).

### 5.1. Increased Risk of HAV Infection

#### 5.1.1. International Travellers

Hepatitis A is one of the most common vaccine-preventable diseases in travellers. It mainly affects people who are unvaccinated and travelling from low endemic areas to high or intermediate endemic countries. Travel-related hepatitis A was responsible for around 46% of all cases in the USA between 2005 and 2007 [44], and for nearly 30% of the reported hepatitis A cases in Europe between 2009 and 2015. The destinations most at risk for European travellers were Turkey, Egypt, and Morocco; they accounted for more than 30% of all hepatitis A infections acquired abroad [45]. Over 40% of the cases declared each year in France involved people who had travelled outside Metropolitan France in the 2 to 6 weeks prior to developing symptoms. The number of cases reported each year peaks between the months of September and October [46], mainly due to infections acquired during July–August, specifically during the summer holidays, in more endemic areas.

Despite the increased risk of HAV infection among travellers and the explicit vaccination recommendations for individuals travelling to countries where infection rates are high or intermediate, vaccination coverage remains low. Fewer than 20% of travellers in the US were vaccinated against hepatitis A in 2018, with large differences between ethnic groups [47,48]. The vaccination rates are also low (<50%) in Japan and several European countries [49,50,51]. This means that both travellers and healthcare workers must become more aware of hepatitis A vaccination recommendations. Airlines and travel companies could make an important contribution by reminding travellers of sanitary risks associated with their destination, as previously suggested [52,53].

#### 5.1.2. Men Who Have Sex with Men (MSM)

Hepatitis A outbreaks among MSM have become quite frequent in developed countries, including European countries, the USA, and Australia [54,55,56,57]. It is now recognized as a sexually transmitted enteric infection, together with shigellosis and salmonellosis [54]. The increased risk of hepatitis A infection among this population led to vaccination being recommended in 1996 [58]. The risk factors most frequently identified in case–control studies were oral–anal and digital–anal intercourse, sex with multiple partners, infection with other sexually transmitted infections (STIs), use of dating apps, and visiting gay saunas and dark rooms [57,59,60,61]. A large outbreak disproportionately affecting MSM occurred in Europe following the Europride festival in Amsterdam, with more than 4000 confirmed cases in 22 European countries [62]. Molecular investigations identified three co-circulating HAV strains belonging to genotype IA (VRD_521_2016, V16–25801, and RIVMHAV16–090) [63]. One strain had been previously described in a large outbreak affecting MSM in 2015 in Taiwan [64,65]. Further outbreaks involving the same lineage and affecting MSM were also reported in the USA and Latin America [66,67]. Worldwide spread of the same strains among the homosexual community highlights the prevalence of international networking in this population and the role of travel in virus dissemination. It also demonstrates the great susceptibility and the low rate of vaccination among MSM; it is estimated to be 25–45% despite the long-lasting recommendations [68,69,70]. More and more MSM have been using HIV pre-exposure prophylaxis (PrEP) to prevent HIV infection since 2016. While several studies reported an increase in traditional STI among people who use PrEP, there are few data available on its influence on HAV incidence. Some French studies have described HAV cases among people who use PrEP at the time when there was a shortage of vaccines in Europe [71,72,73,74]. PrEP initiation and prescription require medical follow-up with regular STI screening. This could encourage vaccination awareness among MSM. Perhaps HAV vaccination should be required in order to access PrEP.

#### 5.1.3. People Who Use Substances and Those Who Face Homelessness

Although hepatitis B and C virus infections are most common among people who use injecting substances, the many outbreaks of acute hepatitis A over the past several decades indicate that this population is also at increased risk of hepatitis A [75,76,77]. While bloodborne infections are possible, this does not seem to be the main route of HAV transmission in this population; people who use non-injecting substances are also at increased risk. Transmission occurs mostly via the faecal–oral route due to inadequate hygiene and living conditions. Case–control studies indicate that HAV infections are associated with a lack of hygiene, such as not washing hands after using the toilet or before preparing food and drugs, sharing needles and syringes, using contaminated drugs, and prior contact with cases of jaundice [76,78]. Outbreaks among people who use substances often result in high fatality rates, probably due to the high percentage of people with chronic hepatitis B or C infections [79]. The main risk factor in a recent outbreak in Indiana involving 264 individuals, 74% of whom were people who use substances, was the use of illicit drugs. This outbreak led to 2% of these individuals dying and 30% contracting acute-on-chronic liver failure [80]. The USA has been affected by a large multistate outbreak of hepatitis A that started in 2016; over 40,000 cases had been reported up to 16 July 2021 [81]. A study of 10% of randomly selected cases from three severely affected states revealed that the morbidity rate was particularly high, with over 50% hospitalized. Many of the people suffering from infection were people who use substances (73.2%) or who face homelessness (14%), and drug use was significantly associated with hospitalization, as well as with being an MSM or experiencing homelessness [82,83,84]. This emphasizes the need to vaccinate these high-risk groups. Another case–control study of an outbreak in San Diego in 2016–2018 showed that the risk of infection, hospitalization, and death due to a hepatitis A infection was 2.5 to 3.9 times greater for people facing homelessness than for people with homes [85]. Vaccination has been recommended for MSM and people who use substances for several decades, and people who face homelessness have recently been added to the Advisory Committee on Immunization Practices (ACIP) list [24]. Although homelessness had been identified as a risk factor for hepatitis A [86,87], the ACIP decided to add people facing homelessness to its vaccination list on October 2018 following these large outbreaks in the USA [88]. Vaccination appears to be the best way to prevent hepatitis A spreading among this population that lacks access to sanitary conditions.

### 5.2. People at Increased Risk of Severe Disease

Several studies have reported that disease severity and fatality rates due to an HAV infection are elevated among people with underlying chronic liver disease, including hepatitis B or C virus coinfection [89], alcoholic cirrhosis, and fatty liver disease [90]. Chronic liver diseases are also frequent in patients with HIV due to coinfections with HBV or HCV, antiviral drug damage, or alcoholic liver diseases. This population could also be at risk of acute-on-chronic liver failure. The high-risk behaviour (oral–anal sex and drug use) of these people results in them being frequently over-represented in HAV outbreaks among MSM [91]. When infected with HAV, patients with HIV may largely contribute to the HAV virus spread as longer HAV viral shedding and higher HAV viral titre have been described in this population [92]. Whereas HAV infections can be more severe in patients with HIV and with liver disease, they have also been associated with less severe forms, with lower peak alanine aminotransferase activities in patients with a plasma RNA load >1000 copies/mL [93,94]. In contrast, patients with HIV had similar clinical outcomes to people without HIV when compliance with antiretroviral therapy was associated with a lower HIV RNA load (<1000 copies/mL) [94].

## 6. Hepatitis A Prevention

No specific treatment is currently available for hepatitis A, only supportive care. Hence, prevention is extremely important. The prevention of all faecal–oral-related diseases is based on improving sanitary and hygienic conditions so as to limit the circulation of the virus and its transmission throughout the community. Safe, effective vaccines have been available since the early 1990s in Europe and the USA, making vaccination the key component of any prevention strategy. Inactive vaccines are the most commonly used but live attenuated vaccines have also been developed in China [22,95,96]. Inactivated vaccines are effective for both pre-exposure and post-exposure prophylaxis and are gradually replacing immunoglobulin-based passive prophylaxis [97,98]. Vaccines have several advantages over immunoglobulins: they induce long-term immunity, and they are inexpensive, easy to administer, and readily available. The use of immunoglobulins is now largely restricted to boosting the vaccination of patients who are immunocompromised and older, as vaccines are less effective in these populations, or as an alternative to vaccination for those for whom the vaccine is contraindicated (people who are allergic and children below 12 months) [99,100].

Post-exposure prophylaxis requires giving inactivated vaccine to all people who are unvaccinated (>12 months) within 2 weeks of exposure. The WHO guidelines for pre-exposure vaccination depend on the local epidemiology. While the vaccine appears to be of limited use in countries where the virus is endemic, because most adults are naturally immunized, the WHO recommends universal vaccination for countries where the infection rate is intermediate. The WHO recommends vaccination for only groups at risk in countries with low and very low infection rates [22]. The universal vaccination of toddlers in many countries has led to significant drops in hepatitis A infection rates both in children and in adults who are unvaccinated [101,102]. The vaccine, alone or combined, is generally given as two to three doses at least 6 months apart. However, a single dose is effective and has been adopted in several countries [103,104]. Whether a single-dose HAV vaccine provides long-lasting immunity still needs further study. Even though HAV vaccine is highly effective, some cases of vaccine escapes with a positive selection of antigenic variants have been reported. This raises the question of new vaccine recommendations for high-risk groups. Some authors have proposed performing a complete vaccine schedule with two doses or to avoid post-exposition vaccine as a prophylaxis to limit the spread of such variants [105].

## 7. Research on HAV Antiviral Treatment

Despite the availability and efficiency of hepatitis A vaccines, the challenge of developing antiviral treatment remains important. These antivirals could prove useful in preventing and treating severe forms or fulminant hepatitis, reducing the duration of symptoms, and shortening the period of infectiveness, thus limiting the risk of outbreaks and the spread of vaccine-escape variants. Research efforts on antivirals directed against the hepatitis A virus have been slowed down due to the commercialization of vaccines, but interesting work has been performed on both host-targeting agents (HTAs) and antivirals [106,107,108].

Regarding broad spectra HTAs, type I interferon (IFN) was found to effectively suppress HAV replication in vitro [109,110] and to improve liver functions in vivo in some cases [111]. Type III IFN, characterized by fewer side effects than type I IFN, could be an interesting alternative as IFN-lambda 1 (IL-29) has been reported to inhibit HAV replication in vitro [112]. Other promising therapeutic targets of HTAs with narrower spectra have been identified, such as the human La protein. La is an RNA-binding protein involved in RNA metabolism. The suppression of La by specific small interfering RNAs (siRNAs) or by janus kinase (JAK) inhibitors (AZD1480, SD-1029, and AG490) leads to the efficient inhibition of HAV replication through the inhibition of HAV internal ribosomal entry-site (IRES) [113,114]. Recent studies have also demonstrated the ability of zinc compounds [115,116,117] and the enzyme heme oxygenase-1 (HO-1) [118], a stress-inducible heat shock protein, to inhibit viral replication in cell culture.

Antiviral drugs used for other viral infections such as ribavirin, amantadine, or sofosbuvir have exhibited antiviral activities against HAV in cell culture systems [119,120,121,122]. In terms of HAV direct-acting antivirals (DAAs), the most attractive targets seem to be the HAV IRES, the 3C protease, and the 3D polymerase. Studies using HAV-specific siRNAs targeting HAV IRES have shown an inhibition of HAV replication in vitro [123]. While there are still few studies on possible polymerase inhibitors [124], many studies have shown the effectiveness of protease inhibitors [125,126,127]. Blocking the entry pathway could also be an interesting strategy, but the identity of HAV cell surface receptors remain to be determined [107].

As most of these studies have been performed on a specific cell line with a specific HAV strain, further investigations are needed on improved HAV cell culture systems and optimal animal models. This will ensure the safety and efficiency of these molecules and further promote the development of new therapeutics.

## 8. Conclusions

HAV infections are a major cause of viral hepatitis worldwide. Globalization and improved sanitary conditions have produced significant changes in HAV epidemiology. Person-to-person transmission, mainly among people at risk, including MSM, people who use substances, and people who face homelessness, is predominant in high-income countries. However, outbreaks still occur despite the availability of safe, effective vaccines, and long-lasting HAV vaccination recommendations for these people. Resources must be developed to promote awareness of HAV among people at high risk and to facilitate their vaccination. Moreover, the development of a specific HAV antiviral treatment could be of great use in containing these outbreaks.
